# Cardioprotective effect of succinate dehydrogenase inhibition in rat hearts and human myocardium with and without diabetes mellitus

**DOI:** 10.1038/s41598-020-67247-4

**Published:** 2020-06-25

**Authors:** Nichlas Riise Jespersen, Marie Vognstoft Hjortbak, Thomas Ravn Lassen, Nicolaj Brejnholt Støttrup, Jacob Johnsen, Pernille Tilma Tonnesen, Steen Larsen, Hans-Henrik Kimose, Hans Erik Bøtker

**Affiliations:** 1 Department of Cardiology, Aarhus University Hospital, Aarhus, Denmark; 2 Xlab, Center for Healthy Aging, Department of Biomedical Sciences, University of Copenhagen, Copenhagen, Denmark; 3 Clinical Research Centre, Medical University of Bialystok, Bialystok, Poland; 4 Department of Cardiothoracic Surgery, Aarhus University Hospital, Aarhus, Denmark

**Keywords:** Cardiovascular biology, Molecular medicine

## Abstract

Ischemia reperfusion (IR) injury may be attenuated through succinate dehydrogenase (SDH) inhibition by dimethyl malonate (DiMAL). Whether SDH inhibition yields protection in diabetic individuals and translates into human cardiac tissue remain unknown. In isolated perfused hearts from 24 weeks old male Zucker diabetic fatty (ZDF) and age matched non-diabetic control rats and atrial trabeculae from patients with and without diabetes, we compared infarct size, contractile force recovery and mitochondrial function. The cardioprotective effect of a 10 minutes DiMAL administration prior to global ischemia and ischemic preconditioning (IPC) was evaluated. In non-diabetic hearts exposed to IR, DiMAL 0.1 mM reduced infarct size compared to IR (55 ± 7% vs. 69 ± 6%, p < 0.05). Mitochondrial respiration was reduced by DiMAL 0.6 mM compared to sham and DiMAL 0.1 mM (p < 0.05). In diabetic hearts an increased concentration of DiMAL (0.6 mM) was required for protection compared to IR (64 ± 13% vs. 79 ± 8%, p < 0.05). Mitochondrial function remained unchanged. In trabeculae from humans without diabetes, IPC and DiMAL improved contractile force recovery compared to IR (43 ± 12% and 43 ± 13% vs. 23 ± 13%, p < 0.05) but in patients with diabetes only IPC provided protection compared to IR (51 ± 15% vs. 21 ± 8%, p < 0.05). Neither IPC nor DiMAL modulated mitochondrial respiration in patients. Cardioprotection by SDH inhibition is possible in human tissue, but depends on diabetes status. The narrow therapeutic range and discrepancy in respiration between experimental and human studies may limit clinical translation.

## Introduction

Attenuation of ischemia reperfusion (IR) injury is fundamental for reducing mortality and morbidity in patients suffering from a myocardial infarction. Ischemic and pharmacological conditioning strategies, as a supplement to revascularization, may reduce IR injury^1^ by ultimately modulating mitochondrial function^2^. While experimental and clinical conditioning studies have shown compelling evidence of infarct size reduction^3,4^, translation into a clinical benefit has been challenging^5,6^. Clinical outcome in patients with ST-elevation myocardial infarction (STEMI) undergoing immediate revascularization has improved significantly^7^. However, the development seems to level out signifying a continuous need for new cardioprotective strategies that go beyond current conditioning modalities in a translational perspective^8^.

During ischemia, hypoxia leads to suppression of the mitochondrial oxidative phosphorylation (OXPHOS)^9^. Revascularization causes a rapid re-establishment of oxygen and consequently mitochondrial OXPHOS. This results in a burst of reactive oxygen species (ROS) emission originating predominantly from complex I in the electron transport chain^10,11^. Succinate increases the complex II (the succinate dehydrogenase (SDH)) linked respiration, which seems to overload the capacity of the electron transport system (ETS), and induce reverse electron transport to complex I and increased ROS production^11^. Increased level of ROS, in combination with mitochondrial damage and dysfunction, initiates a vicious circle of increased mitochondrial dysfunction and further ROS production ultimately causing myocyte death and irreversible myocardial damage^12^.

Mitochondrial metabolism can be modulated to reduce excessive ROS production and limit the myocardial injury^13–15^. Inhibition of the malate aspartate shuttle (MAS) (transport of redox compounds from the cytosol into the mitochondria) protects the myocardium through reduced succinate accumulation and attenuated ROS mediated mitochondrial dysfunction^16^. As SDH is the key enzyme responsible for succinate build-up during ischemia, inhibition of the SDH represents a more targeted approach to reduce succinate levels during ischemia and reperfusion than inhibition of the MAS. Inhibition of SDH protects against IR injury in cardiomyocytes^11^. Studies examining the cardioprotective capacities of SDH inhibition have been conducted in young healthy, non-diabetic animals, so the clinical perspectives of SDH inhibition remain limited.

In patients with diabetes, the outcome following an acute myocardial infarction is impaired compared to patients without diabetes^17^. Compromised outcome in diabetes may be due to metabolic disarrays in the cardiac cells beyond the vascular disease burden^18,19^. Diabetes is associated with mitochondrial dysfunction^20^, even when compared to overweight non-diabetic controls^21^. Additionally, susceptibility to ischemia is impacted by progression of the disease with increased injury in mature diabetes^22^ and the stimulus required for protection increases with duration and severity of the disease^23^. We hypothesized that hearts from mature diabetic individuals have alterations in mitochondrial metabolism leading to mitochondrial dysfunction and an inability to achieve protection by inhibition of the SDH.

We evaluated cardioprotection by pre-ischemic inhibition of the SDH by dimethyl malonate (DiMAL) in animals with or without type 2 diabetes. We transferred these findings into human cardiac tissue by examining the effect of pre-ischemic SDH inhibition by DiMAL on contractile force recovery and mitochondrial function in trabeculae from patients with or without type 2 diabetes.

## Methods

### Ethical approval

The isolated rat heart study conformed to the Danish law for animal research (Act. No. 1306 of 23/11/2007, Danish Ministry of Justice) and the guidelines from *Guide for the Care and Use of Laboratory Animals publishes* by the US National Institutes of Health (NIH Publication No. 85–23, revised 1996). The Danish Animal Experimental Inspectorate approved the experimental work (Authorization No. 2018-15-0201-01446). The human study conformed to the Danish law for clinical studies and the study was approved by the Danish health research ethical committee (Authorization No. 1-10-72-361-15) and registered on clinicaltrials.gov (registration number: NCT02993484). Informed consent was obtained from all participating patients prior to enrollment in the study.

### Biological material

We used 24 weeks old male Zucker diabetic fatty (ZDF) rats (homozygote (fa/fa), n = 57, approximately 400 g, Charles River Laboratories, USA) and age matched non-diabetic controls (heterozygote (fa/+) n = 59, approximately 400 g, Charles River Laboratories, USA) as a model of mature type 2 diabetes following guidelines for rigor and reproducibility in preclinical and clinical studies on cardioprotection^24,25^. Due to excessive food intake the animals developed mature diabetes at 24 weeks of age. Animals were kept at a constant temperature of 23 °C with a 12 hours light-dark cycle and allowed unlimited access to enriched food (Purina 5008; recommended by supplier) and water. No anti-diabetic treatment was given. Rats were fasted 10–12 hours prior to the experiments in order to allow correct measurement of fasting blood glucose levels^22^.

We obtained human cardiac atrial appendage tissue from patients undergoing elective coronary artery bypass grafting (CABG) or valve replacement surgery with the use of extracorporeal circulation. The right atrial appendage was removed to allow insertion of the venous tube into the heart. Following excision of the appendage it was immediately immerged in oxygenated KH buffer (pH 7.35–7.45, room temperature) and transported to the laboratory within 5 minutes.

### Study design

**Figure 1:**
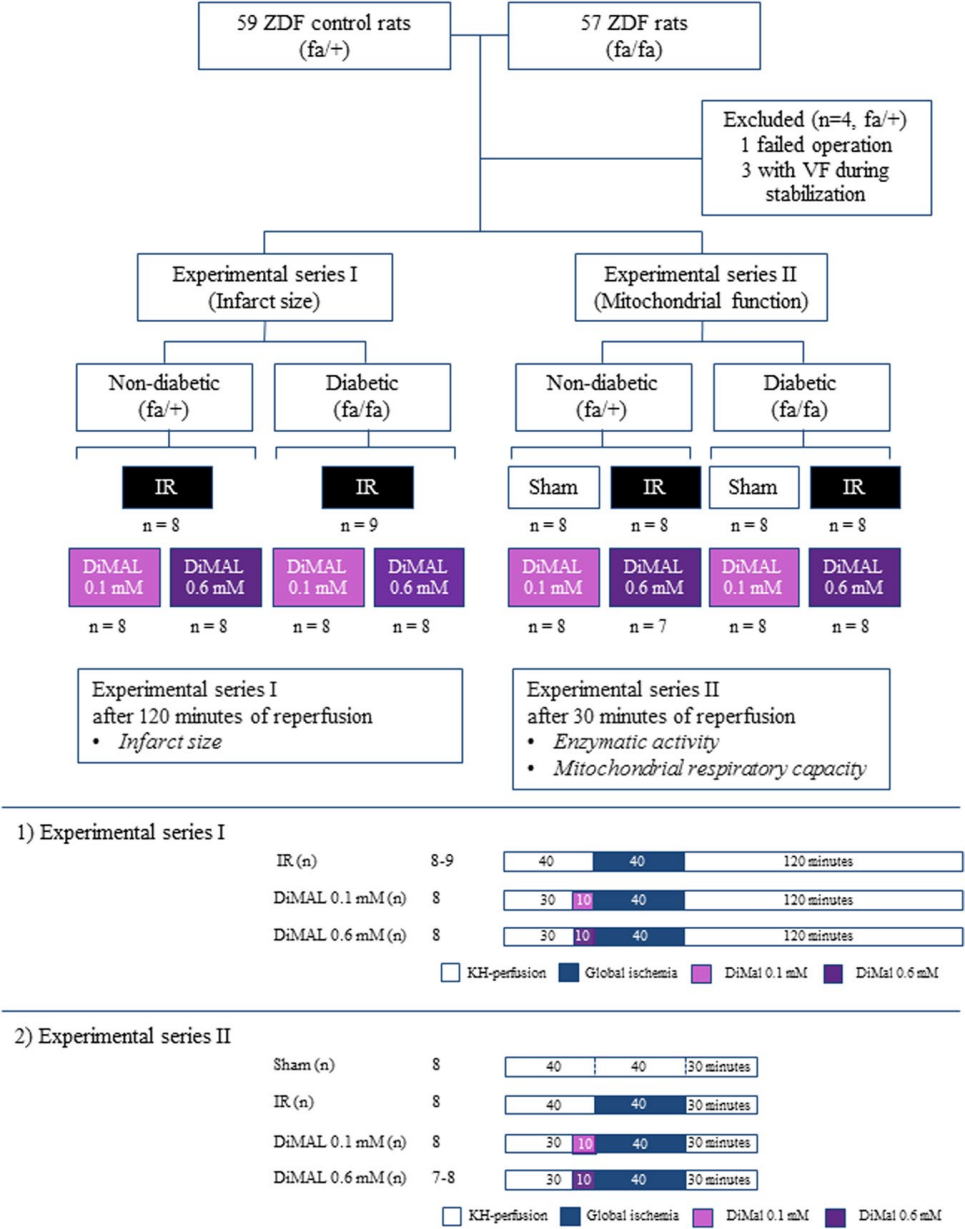
Study design of the isolated rat heart study. An overview of the two experimental series of the isolated rat heart study including subgroups and perfusion protocols. IR: Ischemia reperfusion, DiMAL: Dimethyl Malonate, VF: Ventricular fibrillation.

In the isolated rat heart study, isolated perfused hearts were divided into 4 groups based on the type of interventions (n = 7–9 in each group): (I) Sham hearts (Sham group), (II) IR-injured hearts (IR group) and IR-injured hearts co-perfused with (III) 0.1 mM DiMAL or (IV) 0.6 mM DiMAL for 10 minutes prior to global no-flow ischemia to mimic preconditioning (Fig. 1). All interventions were evaluated in both diabetic and non-diabetic hearts.

We conducted a dose-response correlation and two experimental series: an infarct size study and a mitochondrial respiratory and function study. We did not evaluate infarct size in the Sham groups as it is known to be negligible^26^ and without importance for the conclusions of the study.

Following isolation, hearts were allowed to stabilize for 30–40 minutes depending on the type of intervention. Subsequently, they received 40 minutes global no-flow ischemia followed by 30 minutes (experimental series II) or 120 minutes (experimental series I) of reperfusion. After 30 minutes of reperfusion in experimental series II, the left ventricular muscle was quickly removed and divided into three parts. One part was immediately stored in an ice-cold relaxing solution (BIOPS, composition in mM: 2.77 CaK_2_ EGTA, 7.23 EGTA, 20 taurine, 6.56 MgCl_2_, 5.77 ATP, 15 phosphocreatine, 0.5 dithiothreitol and 50 4-morpholineethanesulphonic acid; pH 7.1) for the measurements of mitochondrial respiratory capacity and mitochondrial fatty acid oxidation. The remaining parts were quickly frozen in liquid nitrogen and stored at −80 °C for later analyses, including mitochondrial enzymatic activities and protein content.

**Figure 2:**
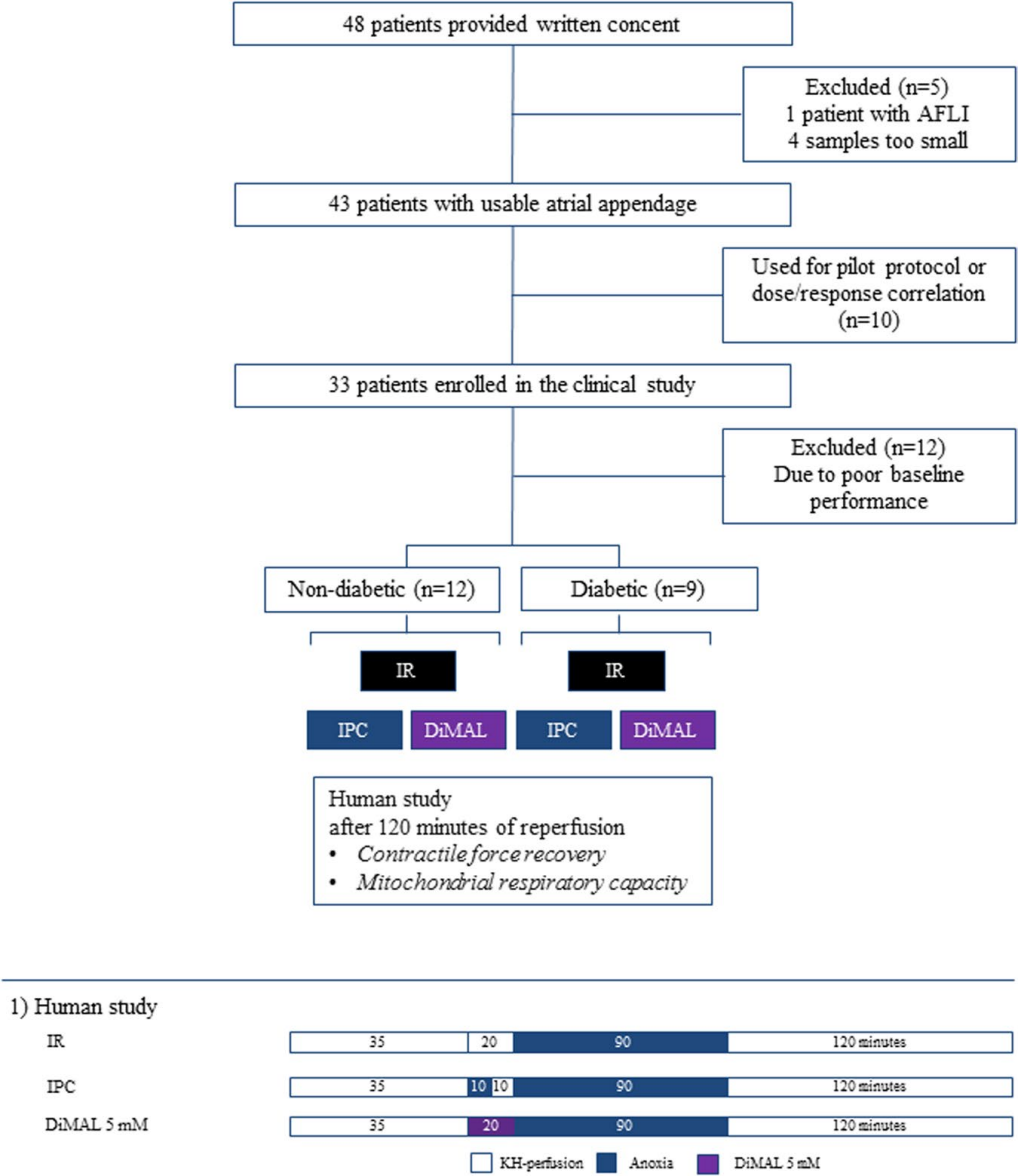
Study design of human study. An overview of the experimental series of the study including subgroups and perfusion protocols. IR: Ischemia reperfusion, IPC: Ischemic preconditioning, DiMAL: Dimethyl Malonate, AFLI: Atrial fibrillation.

In the human study, patients were recruited after project information and giving written consent. The following criteria led to exclusion: age >85 years, atrial fibrillation, ejection fraction <30% or CKMB or Troponin T elevation within 2 weeks. Following excision of the right atrial appendage it was immediately immerged in oxygenated Krebs-Henseleit buffer (pH 7.35–7.45, room temperature) and transported to the laboratory. After suspension in the superperfused atrial strip myograph, each working trabecula was randomized to one of the following groups: (I) IR-injured trabeculae (IR group), (II) IR-injured trabeculae treated with IPC by 10 minutes of anoxia followed by 10 minutes of reperfusion prior to sustained anoxia (IPC group) and (III) IR-injured trabeculae co-perfused with 5 mM DiMAL for 20 minutes prior to sustained anoxia (DiMAL group) (Fig. 2).

### Analysis of blood glucose and plasma metabolites

Prior to isolation of the heart pre-anesthetic fasting blood samples were taken by tail-vein bleeding for measurements of fasting blood glucose (FreeStyle Precision, Abbott Diabetes Care, Copenhagen, Denmark) and plasma insulin as previously described^22^. A fasting blood glucose measurement above 7 mmol/L was used to define the diabetic status. Samples for insulin measurements were collected in heparinized tubes (approximately 400uL) and centrifuged (5000 RPM for 1 minute at ambient temperature). The supernatant was stored at −80 °C before analysis with a hypersensitive rat insulin ELISA kit (DRG instruments, Marburg, Germany). Serum total-cholesterol, triglycerides and free fatty acids were measured in 1 ml blood samples drawn from the abdominal aorta immediately before excision of the heart. Preparations included centrifugation (3500 RPM for 10 minutes at −4 °C), collection of the supernatant and storage at −80 °C until analysis on a Cobas Integra Analyzer (Roche Diagnostics, Rotkreuz, Switzerland).

### Isolated heart preparation

Isolated perfused rat hearts (Langendorff perfusion) were prepared as previously described^27^. Rats were anesthetized by a subcutaneous injection with a mixture of Dormicum (midazolam, 0.5 mg (kg body weight^−1^); Matrix Pharmaceuticals, Herlev, Denmark) and Hypnorm (fentanyl citrate, 0.158 mg (kg body weight^−1^ and fluanisone, 0.5 mg (kg body weight^−1^)).

A tracheotomy was performed and the rat was connected to a rodent ventilator (Ugo Basile 7025 rodent ventilator, Comerio, Italy) and ventilated at 60 breaths/minute with a tidal volume of 3 mL. Subsequently, a laparotomy and thoracotomy were performed and a bolus of 1000 IU/kg heparin (Leo Pharma, Ballerup, Denmark) was administrated through the femoral vein. The ascending aorta was cannulated *in situ* and retrograde perfusion of the heart was commenced at a constant pressure of 80 mmHg with an oxygenated (95% O_2_ and 5% CO_2_) Krebs Henseleit buffer (KHB: composition in mM: NaCl 118.5, KCl 4.7, NaHCO_3_ 25.0, glucosemonohydrate 11.0, MgSO_4_·7H_2_O 1.2, CaCl_2_ 2.4 and KH_2_PO_4_ 1.2). The heart was excised under continuous perfusion and mounted in an isolated perfused heart system (IH-SR type844/1; HSE, March-Hugstetten, Germany) where temperature is kept constant at 37 °C. An intraventricular balloon (Size 7, HSE, March-Hugstetten, Germany) was inserted in the left ventricle after removing the left atrial appendage and the intraventricular balloon volume was adjusted to a left ventricular end-diastolic pressure of 4–8 mmHg to simulate preload. Coronary flow was monitored continuously using an inline flow probe (Type 2.5SB, Transonic System Inc., Ithaca, NY, USA). Data was digitally converted (DT9804; Data Translation. Marlboro, MA, USA) and stored using Notocord Hem software (version 2.0, Notocord systems, Croissy sur Seine, France).

### Atrial strip model

Isolated atrial trabeculae were prepared as previously described^28^. Patients undergoing CABG or valve-replacement surgery were enrolled in the study. Within 20 minutes of excision of the atrial appendage, atrial trabeculae were tied by a suture (4.0 silk) in each end, excised from the remaining atrial appendage and mounted in the atrial strip myograph. Sutures are connected to a force transducer and a fixed hook.

The atrial trabeculae (length ranging between 5 and 10 mm and diameter ranging between 400–600 µm) were submerged into 37 °C Krebs-Henseleit buffer oxygenated with 95% O_2_ and 5% CO_2_ to maintain pH between 7.35 and 7.45. The isolated trabeculae were stimulated with 1 Hz by platinum electrodes connected to stimulators (Harvard Apparatus, amplitude 30 V, duration 1 ms) during stabilization and reoxygenation. Simulated ischemia was established by increased pacing (3 Hz) and changing to a hypoxic buffer (composition in mM: NaCl 118.5, KCl 4.7, NaHCO_3_ 25.0, choline chloride 14.0, MgSO_4_·7H_2_O 1.2, CaCl_2_ 2.4 and KH_2_PO_4_ 1.2), which was deoxygenated with 95% N_2_ and 5% CO_2_. After mounting the trabeculae in the organ baths, all trabeculae were stretched passively to a pretension of 0.5 g and left to stabilize for 30 minutes. Subsequently pretension was increased to 0.75 g for additional 45 minutes of stabilization.

### Infarct size

At the end of perfusion in experimental series I of the isolated rat heart study, hearts were frozen at −80 °C, sliced (1.5 mm thick) and vital stained with 1% 2,3,5-triphhenyltetrazoliumchloride (Merck, Darmstadt, Germany) for 3 minutes at 37 °C to delineate areas of infarction. The stained heart slices were preserved in formaldehyde (10%) for 24–48 hours and then scanned using a flatbed scanner (Epson Perfection V600, Epson, Suwa, Japan) and weighed (Mettler-Toledo level balances, Mettler-Toledo, USA)^16^. Area-at-risk and area-of-infarction (infarct size) were assessed using image analysis software (ImageJ, National Institute of Health, USA) and weighted with the wet weight of the individual slices. Finally, infarct size/area-at-risk (IS/AAR) ratio was calculated. An observer blinded to treatment and disease groups analyzed the data.

### Contractile force assessment

Contractile force was measured using a force transducer and data was digitally converted (DT9804; Data Translation. Marlboro, MA, USA) and stored using Notocord Hem software (version 2.0) (Notocord systems, Croissy sur Seine, France). Contractile force recovery was calculated as a ratio of post-ischemic contractile force divided by pre-ischemic contractile force and served as a surrogate marker of myocardial IR injury^28^.

### Mitochondrial respiratory capacity

At the end of the perfusion protocol in experimental series II of the isolated rat heart study, the left ventricle was quickly divided in two samples during continued perfusion for subsequent measurement of mitochondrial respiration and various tissue analyses. In the human study the trabeculae were removed and the areas affected by the ligature were discarded. The tissue was stored temporarily in a cold (0–4 °C) relaxation buffer (BIOPS; composition in mM: CaK_2_EGTA 2.77, K_2_EGTA 7.23, Na_2_ATP 5.77, MgCl_2_·6H_2_O 6.56, Taurine 20, Na_2_Phosphocreatine 15, Imidazole 20, Dithiothreitol 0.5 and MES 50; pH 7.1; kept between 0–4 C°) until isolation of the fibers.

Mitochondrial respiration was measured in permeabilized cardiac muscle fibers as previously described^16^. Muscle fibers were dissected free of connective tissue in ice-cold BIOPS buffer using sharp forceps. After dissection, fibers were placed in ice-cold BIOPS-buffer supplemented with 50 μg·mL^−1^ Saponin for 30 minutes to ensure permeabilization. Fibers were subsequently washed by agitation in ice-cold MiR05-buffer (composition in mM): EGTA 0.5, MgCl_2_·6H_2_O 3.0, K-lactobionate 60, Taurine 20, KH_2_PO_4_ 10, HEPES 20, Sucrose 110 and BSA 1 g·L^−1^; pH 7.1) for two times 10 minutes. Muscle fibers were then weighed and transferred to an oxygraph (Oxygraph-2k; Oroboros, Innsbruck, Austria) for measurement of glucose supported metabolism (protocol l) and fatty acid supported metabolism (protocol 2). All measurements were performed as duplicates in hyperoxygenation to avoid potential oxygen limitations to respiration. Due to limited tissue available for high-resolution respirometry only protocol 1 was applied in the human study.

Protocol 1 (complex I + II-linked respiration): Malate (2 mM) and glutamate (10 mM) were added to evaluate state 2 respiration (GM). After addition of ADP (5 mM) state 3 respiration was achieved (GM3). Respiratory control ratio (RCR) was calculated as state 3/state 2. Cytochrome *c* (10 μM) was added to evaluate outer mitochondrial membrane integrity (more than 10% increases in respiration lead to exclusion). Subsequently, succinate (20 mM) was added to activate complex II during state 3 (GMS3) followed by Oligomycin (2.5 μM) to achieve state 4 respiration (4o). Carbonyl cyanide-p-trifluoromethoxyphenylhydrazone (FCCP) was added to evaluate maximal respiratory capacity in a non-coupled state (E). Finally, Rotenone (0.5 μM) and Antimycin A (2.5 μM) were added to estimate residual oxygen consumption (ROX).

Protocol 2 (fatty acid oxidation): Malate (2 mM) was added to give fundamental stimulation of complex I (state 2 respiration) followed by multiple titrations of Octanoyl-1-carnitine to achieve maximal β-oxidation and complex II respiration (MOc). State 3 respiration is achieved by adding ADP (5 mM, MOc3) and finally cytochrome c (10 μM) was added to examine outer membrane integrity.

### Mitochondrial enzyme activity

Citrate synthase (CS) activity, a key enzyme in the glucose oxidation and marker of mitochondrial content, and *β*-hydroxyacyl-CoA dehydrogenase (HAD) activity, a key enzyme in the fatty acid oxidation, were measured in cardiac tissue homogenate by spectrophotometry as previously described^29^. In short, 2–3 mg wet weight of cardiac muscle were homogenized in a TissueLyser (Qiagen, Venlo, Limburg, The Netherlands). For CS, the homogenate was diluted 50 times and the conversion of 5.5-dithiobis-(2-nitrobenzoic acid) to 5-thiobis-(2-nitrobenzoic acid) was measured at 37 °C spectrophotometrically at 415 nm on an automatic analyzer (Cobas 6000, C 501, Roche Diagnostics, Mannheim, Germany). For HAD, the homogenate was diluted 70 times and changes in NADH at 37 °C were measured spectrophotometrically at 340 nm on an automatic analyzer (Cobas 6000, C 501, Roche Diagnostics).

### Mitochondrial protein content of ETC complexes

Mitochondrial protein content was measured as previously described^30^. In short, the protein homogenates were diluted in Laemmeli buffer and 15 µg of total protein from each sample was separated on precast criterion gels (Bio-Rad, Copenhagen, Denmark). After electrophoresis, the gel was electrophoretically transferred to a polyvinylidene fluoride (PVDF) membrane. The membranes were blocked over night at 4 °C with skim milk diluted in phosphate-buffered saline. The Total OXPHOS antibody cocktail (Abcam, ab110413) was used to determine the proteins of interest and set to incubate at room temperature for 1 hour followed by incubation with horseradish peroxidase-conjugated secondary antibodies (Dako, Glostrup, Denmark). Visualization of antibody-specific labeling bands were revealed with activation with enhanced chemiluminescence Western blotting detection system (ECL, GE Healthcare, Little Chalfont, UK) and a CCD camera (LAS 4000, GE Healthcare). We quantified intensities of each specific band obtained by chemiluminescence using Image Quant TL software (GE Healthcare, USA) and normalized to the entire UV intensity of the corresponding sample representing the total protein content on the membrane.

### Complex II activity

Frozen tissue was homogenized in a PBS buffer supplemented with protease inhibitors and prepared for complex II activity measurements using a Complex II Enzyme activity assay kit (Abcam, ab109908). Colorimetric measurements were achieved using PheraStar FS (BMG Labtech, Aylesbury, UK).

#### Myocardial interstitial concentrations of succinate

Myocardial interstitial concentration of succinate was measured in the 0.6 mM DiMAL group in non-diabetic and diabetic rats by microdialysis as previously described^27^. A microdialysis probe (membrane length 4 mm, cut-off 6 Da; AgnTho’s, Lidingoe, Sweden) was inserted into the free wall of the left ventricle. The microdialysis probe was continuously perfused at a flow speed of 1 μL/minutes. with Krebs-Henseleit buffer solution deoxygenated with 95% N_2_ and 5% CO_2_. Following the implantation, a 20 minutes stabilization period was allowed for the metabolites to reach equilibrium in the perturbed tissue. Samples were collected at 10 minutes intervals and later analyzed by liquid chromatography and mass spectrometry.

### Statistical analysis

All results are expressed as mean ± SD. Non-diabetic and diabetic groups were compared by two-way ANOVA. Comparison between baseline characteristics of patients with and without diabetes was done by t-test. Between groups comparison was performed using one-way ANOVA followed by a Bonferroni post-hoc analysis when appropriate. We used Prism, version 6.0 (GraphPad Software, La Jolla, CA, USA) for all statistical analyses. *P* < 0.05 was considered statistically significant.

## Results

### Exclusions

#### Isolated rat heart study

A total of 4 rats (all non-diabetic) were excluded prior to enrolment in the study (Fig. 1). Three rats were excluded due to ventricular fibrillation in the stabilization period and one rat due to unsuccessful surgical procedure. Complete datasets were achieved from the remaining rats.

#### Human study

In the human study 5 patients were excluded after giving written consent due to small atrial tissue sample sizes (Fig. 2). 10 patients were allocated to protocol optimization and dose/response correlation. 33 patients were enrolled in the study and 12 patients were excluded because the fibers did not reach sufficient hemodynamic performance of 0.5 g of contractility at the end of stabilization^28^.

### Baseline characteristics

#### Isolated rat heart study

Bodyweight, heart weight, fasting blood glucose levels and biochemical characteristics are summarized in Table 1. Body and heart weight did not differ between diabetic and non-diabetic rats. Fasting blood glucose was significantly higher in diabetic groups compared to the respective non-diabetic groups. Diabetic rats in the DiMAL 0.1 mM groups had significantly higher glucose levels than the other diabetic groups. We found no significant differences between all other diabetic and non-diabetic disease groups.

**Table 1:** Characteristics of the ZDF rat model. Data are mean ± SD. Ŧ P < 0.05 grouped diabetic rats (fa/fa) compared to their grouped age-matched controls. ^#^P < 0.05 diabetic group vs. diabetic DiMAL 0.1 mM.

Type		Non-diabetic	Non-diabetic	Non-diabetic		Diabetic	Diabetic	Diabetic
Group		IR (n = 8)	DiMAL 0.1 mM (n = 8)	DiMAL 0.6 mM (n = 8)		IR (n = 9)	DiMAL 0.1 mM (n = 8)	DiMAL 0.6 mM (n = 8)
Characteristics of the ZDF rat model								
Infarct size study								
Body weight (BW), g		402 ± 22	405 ± 30	404 ± 25		403 ± 37	407 ± 28	387 ± 21
Heart weight (HW), g		1.18 ± 0.15	1.20 ± 0.10	1.23 ± 0.12		1.23 ± 0.11	1.18 ± 0.07	1.19 ± 0.05
HW/BW ratio		0.29 ± 0.03	0.30 ± 0.02	0.30 ± 0.02		0.31 ± 0.03	0.29 ± 0.02	0.31 ± 0.02
B-glucose, mmol/L		4.7 ± 0.4	5.5 ± 0.7	4.7 ± 0.3		15.0 ± 4.1 Ŧ^#^	24.7 ± 1.9 Ŧ	12.8 ± 3.4 Ŧ^#^
P-insulin, pmol/L		44.2 ± 18.1	47.9 ± 9.7	41.2 ± 15.4		88.0 ± 27.6 Ŧ	89.4 ± 21.7 Ŧ	82.7 ± 14.3 Ŧ
S-total cholesterol, mmol/L		2.17 ± 0.20	2.46 ± 0.27	2.29 ± 0.40		4.11 ± 0.72 Ŧ^#^	5.18 ± 077 Ŧ	4.00 ± 0.46 Ŧ^#^
S-triglyceride, mmol/L		0.88 ± 0.52	0.78 ± 0.32	0.97 ± 0.39		9.81 ± 1.50 Ŧ	8.87 ± 0.53 Ŧ	9.90 ± 0.56 Ŧ
S-free fatty acids, mmol/L		1.70 ± 1.34	1.80 ± 0.78	1.71 ± 0.58		6.14 ± 0.56 Ŧ	5.83 ± 1.49 Ŧ	5.21 ± 1.77 Ŧ
Mitochondrial function study								
**Type**	**Non-diabetic**	**Non-diabetic**	**Non-diabetic**	**Non-diabetic**	**Diabetic**	**Diabetic**	**Diabetic**	**Diabetic**
**Group**	**Sham (n** = **8)**	**IR (n** = **8)**	**DiMAL 0.1** **mM (n** = **8)**	**DiMAL 0.6** **mM (n** = **7)**	**Sham (n** = **8)**	**IR (n** = **9)**	**DiMAL 0.1** **mM (n** = **8)**	**DiMAL 0.6** **mM (n** = **8)**
Body weight (BW), g	413 ± 26	397 ± 18	400 ± 34	423 ± 31	408 ± 46	370 ± 38	390 ± 19	392 ± 42
B-glucose, mmol/L	4.8 ± 0.2	5.0 ± 0.4	5.1 ± 0.5	4.7 ± 0.3	12.5 ± 3.1 Ŧ^#^	13.0 ± 2.7 Ŧ^#^	24.2 ± 4.0 Ŧ	11.9 ± 1.8 Ŧ^#^
P-insulin, pmol/L	47.2 ± 16.2	36.4 ± 12.9	49.4 ± 10.1	46.3 ± 12.1	82.0 ± 24.8 Ŧ	75.3 ± 18.0 Ŧ	74.3 ± 18.0	83.1 ± 32.8 Ŧ
S-total cholesterol, mmol/L	2.19 ± 0.15	2.23 ± 0.18	2.09 ± 0.57	2.43 ± 0.14	4.32 ± 0.32 Ŧ	3.83 ± 0.75 Ŧ^#^	4.95 ± 0.95 Ŧ	4.04 ± 0.27 Ŧ^#^
S-triglyceride, mmol/L	0.94 ± 0.44	0.94 ± 0.37	0.87 ± 0.21	1.18 ± 0.42	9.97 ± 0.77 Ŧ^#^	10.14 ± 0.53 Ŧ^#^	8.37 ± 0.96 Ŧ	9.99 ± 0.81 Ŧ^#^
S-free fatty acids, mmol/L	1.73 ± 0.72	1.50 ± 0.90	1.83 ± 0.51	2.03 ± 1.14	5.92 ± 0.78 Ŧ	5.18 ± 2.07 Ŧ	4.71 ± 1.45 Ŧ	5.063 ± 1.51 Ŧ

The diabetic groups had significant elevation in the biochemical variables, including insulin, cholesterol, triglycerides and free fatty acids (Table 1), compared to their respective non-diabetic groups. S-cholesterol was significantly elevated in DiMAL 0.1 mM compared to other diabetic groups. Between all other groups within the diabetic and non-diabetic disease groups, we found no significant differences.

#### Human study

We found no differences between patients with and without diabetes on age, BMI, total cholesterol, LDL-, HDL-cholesterol or triglyceride (Table 2). We found significantly higher HbA1c levels in patients with diabetes compared to patients without diabetes (8.7 ± 1.0 vs. 6.2 ± 0.6 mmol/L of HbA1c, P < 0.05). All patients with diabetes received antihypertensive and statin treatment, compared to only 58% and 25% in patients without diabetes (p < 0.05).

**Table 2:** Patient characteristics. Data are mean ± SD.

Patients characteristics			
Type	Patient without diabetes	Patient with diabetes	p value
	(n = 12)	(n = 9)	
Age, year (mean ± SD)	64 ± 9	63 ± 7	0.75
Male gender, n (%)	11 (92)	8 (77)	0.78
Smoker, n (%)	1 (8)	1 (11)	0.78
Former smoker, n (%)	7 (58)	6 (67)	0.47
BMI, kg/m^2^ n (mean ± SD)	27.1 ± 4.4	30.0 ± 4.0	0.15
HbA1C, mmol/L (mean ± SD)	6.2 ± 0.6	8.7 ± 1.0	**<0.05**
Total cholesterol, mmol/L (mean ± SD)	4.0 ± 1.0	4.3 ± 1.4	0.63
LDL, mmol/L (mean ± SD)	2.2 ± 0.7	2.5 ± 1.1	0.41
HDL, mmol/L (mean ± SD)	1.1 ± 0.3	0.9 ± 0.2	0.30
Triglyceride, mmol/L (mean ± SD)	1.7 ± 1.0	1.8 ± 0.5	0.70
Statin therapy, n (%)	3 (25)	9 (100)	**<0.05**
Antihypertensive therapy, n (%)	7 (58)	9 (100)	**<0.05**

### Infarct size

#### Isolated rat heart study - Dose-response relationship

**Figure 3:**
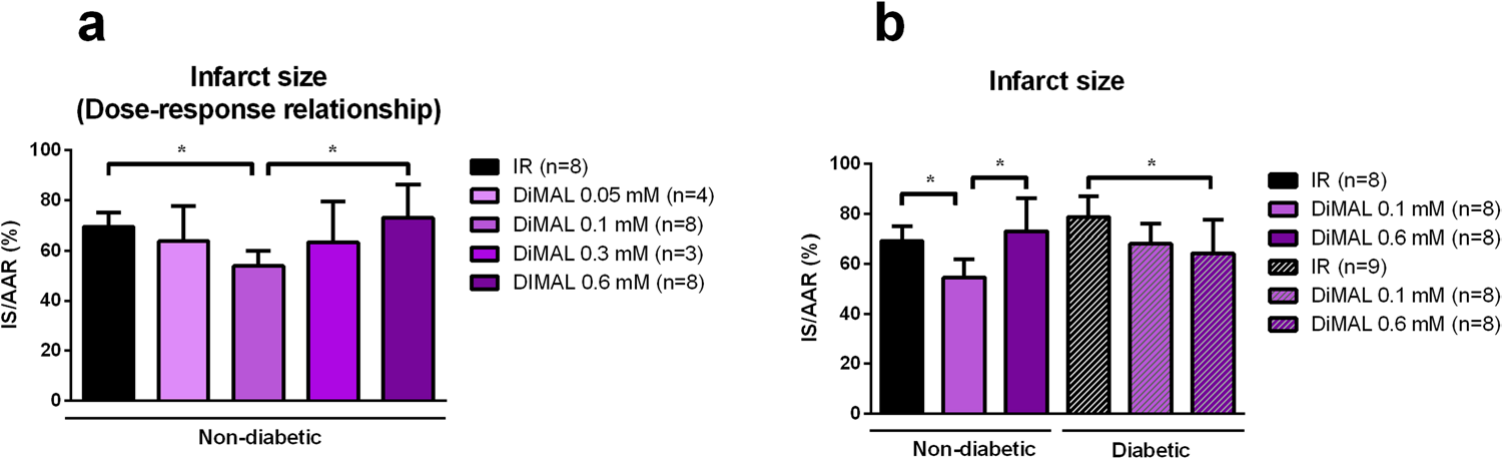
Isolated rat heart study – Dose-response relationship and infarct size. Dose-response relationship showing the effect of DiMAL on infarct size (IS) as a percentage of area at risk (AAR) in non-diabetic rat hearts (**a**) and IS/AAR of different intervention groups in non-diabetic and diabetic ZDF rat hearts (**b**). IR: Ischemia reperfusion, DiMAL: Dimethyl Malonate. Data are the mean ± SD. *P < 0.05 between indicated groups.

The dose-response relationship of DiMAL treatment in non-diabetic rat hearts revealed a u-shaped relationship between dose and infarct size. Infarct size was reduced in the 0.1 mM DiMAL group compared to IR alone (54 ± 6% vs. 69 ± 6% of LV, p < 0.05) (Fig. 3). DiMAL concentrations of 0.05 mM, 0.3 mM and 0.6 mM did not provide reductions in infarct size compared to IR (64 ± 14%, 63 ± 16% and 73 ± 13% vs. 69 ± 6%).

#### Isolated rat heart study - infarct size

Infarct size following IR did not differ statistically significantly in diabetic compared to non-diabetic rats (79 ± 8% and 69 ± 6%, p = 0.07) (Fig. 3). In non-diabetic animals, DiMAL 0.1 mM reduced infarct size compared to the IR group (55 ± 7% vs. 69 ± 6%, p < 0.05). DiMAL 0.6 mM yielded no protection (73 ± 13% and 69 ± 6%, p = 0.71) (Fig. 3). In diabetic hearts, DiMAL 0.1 mM treatment did not reduce infarct size compared to the IR group (68 ± 8% and 79 ± 8%, p = 0.14) whereas DiMAL 0.6 mM yielded significant protection (64 ± 13% vs. 79 ± 8%, p < 0.05).

### Contractile force recovery

#### Human study - Dose-response relationship

**Figure 4:**
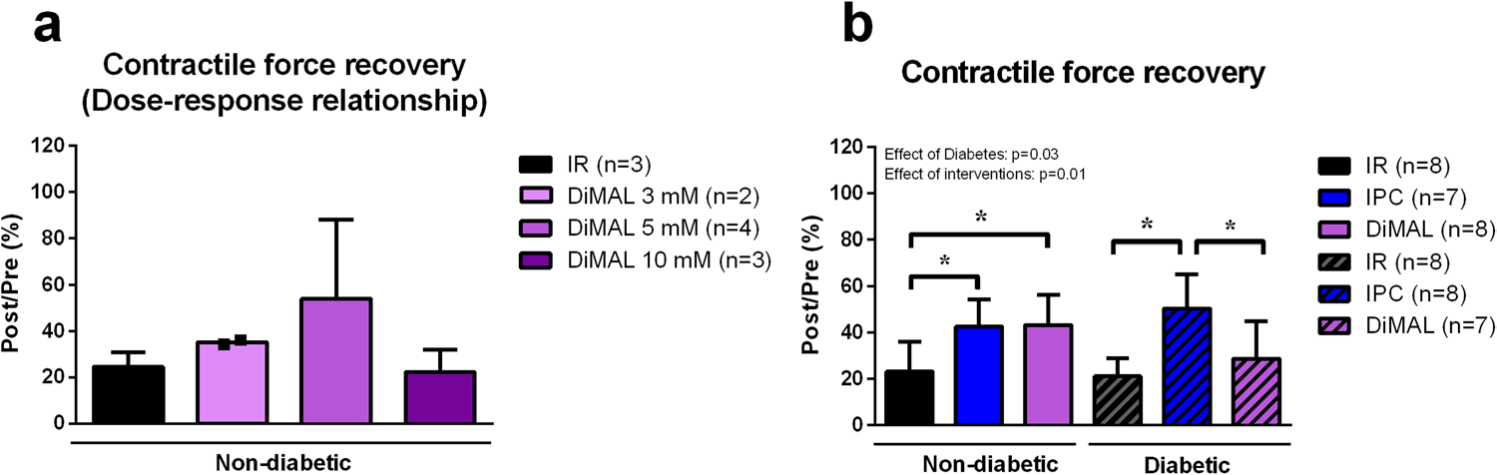
Human study – Dose-response relationship and contractile force recovery. Dose-response relationship between DiMAL and contractile force recovery in non-diabetic human atrial trabeculae (**a**) and contractile force recovery of non-diabetic and diabetic human atrial trabeculae given as a ratio of contractile force in the reperfusion compared to the pre-ischemic value (**b**). IR: Ischemia reperfusion, DiMAL: Dimethyl Malonate, IPC: Ischemic preconditioning. Data are mean ± SD. *P < 0.05 between indicated groups. P-values at the top indicate overall effect of diabetes and interventions by two-way ANOVA, respectively.

The dose-response relationship of DiMAL treatment in non-diabetic human atrial trabeculae showed that a concentration of 5 mM yielded the largest improvement in contractile force recovery compared to IR alone (54 ± 34% vs. 24 ± 6% of contractile force recovery, p = 0.67) (Fig. 4). Reduction of DiMAL concentration to 3 mM or an increment to 10 mM attenuated or abolished contractile force recovery improvements, respectively, compared to IR alone (35 ± 1%, 22 ± 10% and 24 ± 6% of contractile force recovery, p > 0.99).

#### Human study

Contractile force did not differ between patients without and with diabetes (ANOVA p > 0.99). IPC improved contractile force recovery in trabeculae from patients without diabetes compared to trabeculae undergoing IR alone (43 ± 12% vs. 23 ± 13%, p < 0.05) (Fig. 4). DiMAL 5 mM yielded the same improvement in non-diabetic trabeculae (43 ± 13% vs. 23 ± 13%, p < 0.05). IPC in trabeculae from patients with diabetes improved contractile force recovery compared to IR and DiMAL (51 ± 15% vs. 21 ± 8% and 29 ± 16%, p < 0.05). In contrast, DiMAL did not significantly affect contractile force recovery compared to IR in diabetic trabeculae (29 ± 16% and 21 ± 8%, p = 0.83).

### Mitochondrial respiratory capacity

#### Isolated rat heart study

**Figure 5:**
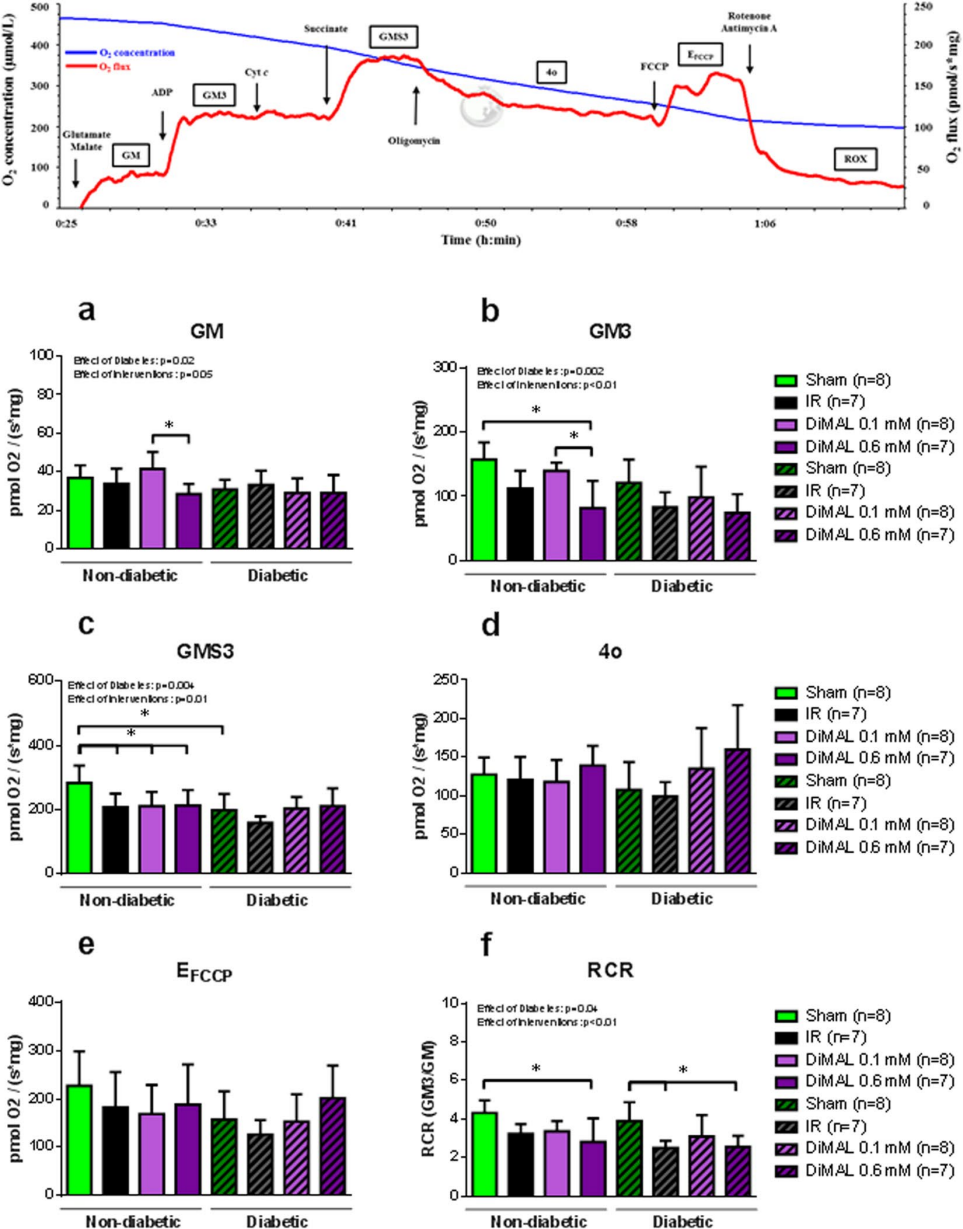
Isolated rat heart study - Mitochondrial respiratory capacity. Representative graph of mitochondrial respiratory capacity from a Sham heart. Below mitochondrial respiratory capacity. GM, state 2 respiration with glutamate + malate (**a**); GM3, state 3 respiration with glutamate and malate (**b**); GMS3, state 3 respiration with glutamate, malate and succinate (**c**); 4o, state 4 respiration with oligomycin (**d**); FCCP, non-coupled state by FCCP (**e**); RCR with complex I-linked substrates (**f**). Data are mean ± SD. *P < 0.05 between indicated groups. P-values at the top indicate overall effect of diabetes and interventions by two-way ANOVA, respectively.

Diabetes reduced mitochondrial respiration capacity compared to non-diabetic hearts as evaluated by GM, GM3, GMS3 and RCR (Fig. 5, ANOVA: p = 0.02, p = 0.002, p = 0.004 and p = 0.04, respectively). DiMAL 0.1 mM increased State 2 respiration (GM) compared to DiMAL 0.6 mM but not compared to IR in non-diabetic hearts (41.6 ± 8.7 vs. 28.5 ± 5.3 and 33.9 ± 7.9 pmol O_2_/(s*mg), p < 0.05 and p = 0.25). We observed no other differences in state 2 respiration in either the non-diabetic or the diabetic group. State 3 complex I-linked respiration capacity (GM3) was higher in both the Sham and DiMAL 0.1 mM groups than in the DiMAL 0.6 mM group in non-diabetic hearts (157.4 ± 26.5 and 140.0 ± 35.3 vs. 81.8 ± 42.4 pmol O_2_/(s*mg), p < 0.01 and p < 0.05). IR alone numerically reduced the respiration compared to Sham (157.4 ± 26.5 vs. 112.3 ± 27.0 pmol O_2_/(s*mg), p = 0.09). State 3 complex I- and II-linked respiration (GMS3) was higher in non-diabetic Sham hearts than in non-diabetic hearts exposed to IR, DiMAL 0.1 mM, DiMAL 0.6 mM as well as in the diabetic Sham group (283.5 ± 53.6 vs. 207.6 ± 42.6, 211.0 ± 44.1, 213.3 ± 48.0 and 198.8 ± 50.0 pmol O_2_/(s*mg) (p < 0.05), respectively). The RCR was higher in the Sham groups compared to any of the intervention groups regardless of the presence of diabetes. No differences were observed between intervention groups in state 4o (Fig. 5), non-coupled state E with FCCP (Fig. 5) or ROX (data not shown).

#### Human study

**Figure 6:**
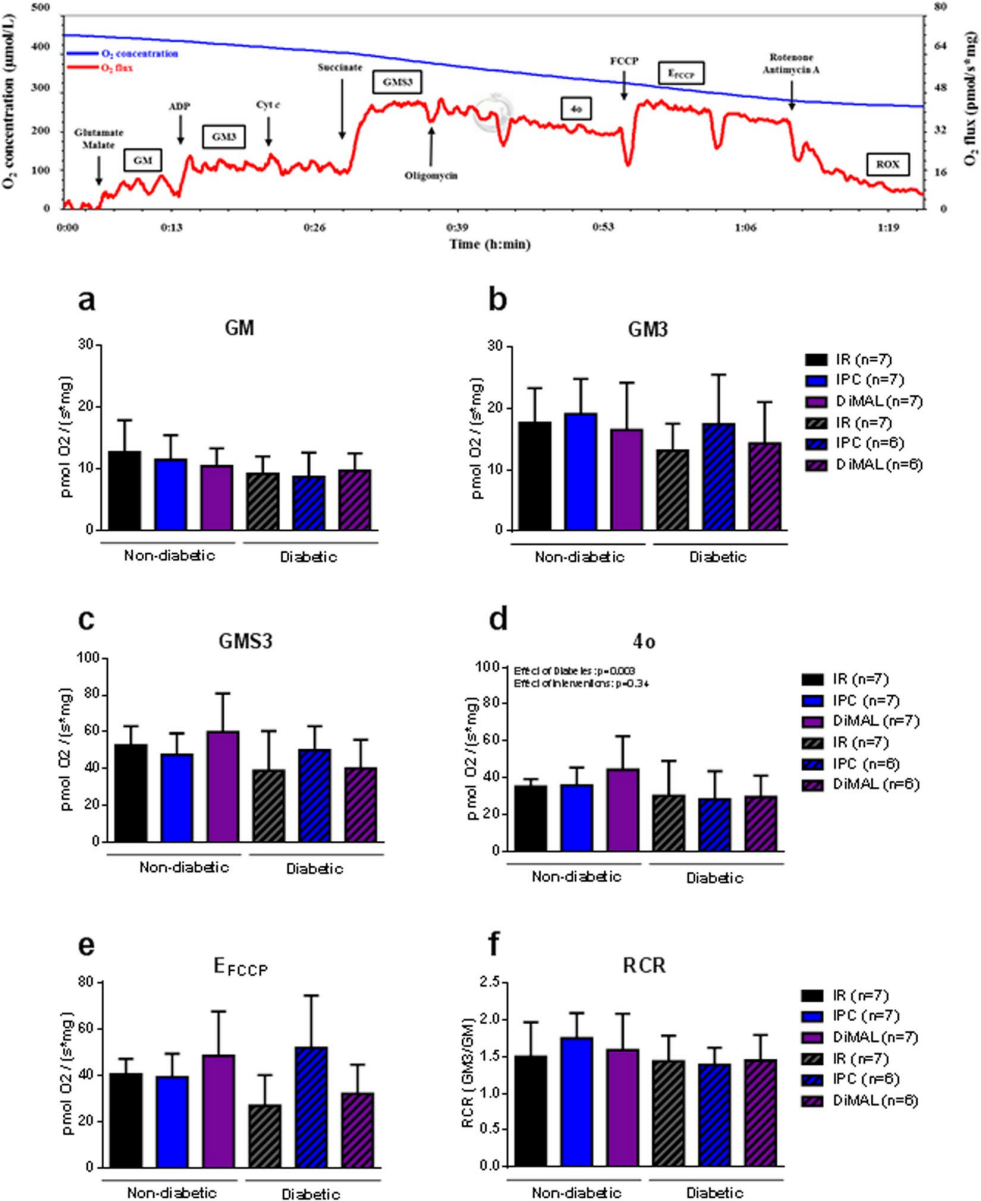
Human study - Mitochondrial respiratory capacity. Representative graph of mitochondrial respiratory capacity from an IR heart trabecula. Below mitochondrial respiratory capacity. GM, state 2 respiration with glutamate + malate (**a**); GM3, state 3 respiration with glutamate and malate (**b**); GMS3, state 3 respiration with glutamate, malate and succinate (**c**); 4o, state 4 respiration with oligomycin (**d**); FCCP, non-coupled state by FCCP (**e**); RCR with complex I-linked substrates (**f**). Data are mean ± SD. *P < 0.05 between indicated groups. P-values at the top indicate overall effect of diabetes and interventions by two-way ANOVA, respectively.

State 2 respiration (GM) and state 3 complex I-linked respiration (GM3) did not differ between trabeculae from patients with and without diabetes and was not affected by IPC or DiMAL (Fig. 6). Also, State 3 complex I + II linked respiration (GMS3) further increased the level of respiration in all groups but with no differences between trabeculae from patients with and without diabetes or between interventions. Consistent with this finding, we observed no change in RCR by any intervention. State 4o, non-coupled state E with FCCP and ROX were similar in all intervention groups and between trabeculae from patients with and without diabetes (ROX data not shown).

### Mitochondrial fatty acid oxidation

#### Isolated rat heart study

**Figure 7:**
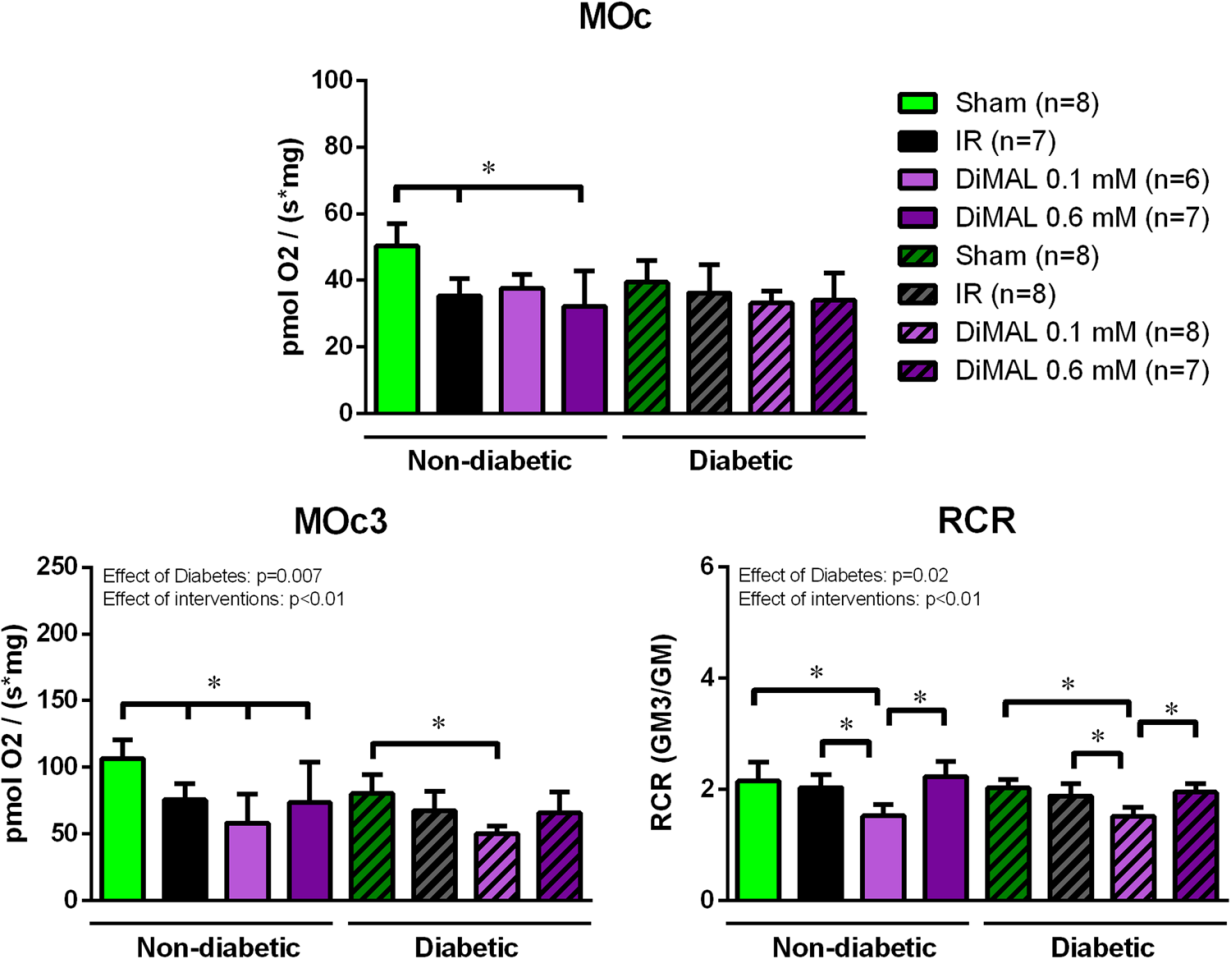
Isolated rat heart study - Mitochondrial fatty acid respiratory capacity. Summarized data of mitochondrial fatty acid respiratory capacity in each respiratory state. MOc, state 2 respiration with malate and octanoyl-l-carnitine; MOc3, state 3 respiration with malate and octanoyl-l-carnitine; RCR with fatty acids. Data are the mean ± SD. *P < 0.05 between indicated groups. P-values at the top indicate overall effect of diabetes and interventions by two-way ANOVA, respectively.

Diabetes reduced mitochondrial respiration capacity compared to non-diabetic hearts as evaluated by MOc3 and RCR (Fig. 7, ANOVA: p = 0.007 and p = 0.02). All interventions reduced mitochondrial fatty acid oxidation compared to the non-diabetic sham group. (MOc: Sham 50.3 ± 6.7 vs. IR 35.4 ± 5.2 vs. DiMAL 0.1 mM 37.7 ± 10.2 vs. DiMAL 0.6 mM 32.2 ± 10.6 pmol O_2_/(s*mg) (p < 0.05); MOc3: Sham 106.6 ± 14.0 vs. IR 75.5 ± 12.0 vs. DiMAL 0.1 mM 58.2 ± 21.6 vs. DiMAL 0.6 mM 73.6 ± 30.2 pmol O_2_/(s*mg)) (p < 0.05) (Fig. 7). Pre-ischemic inhibition by DiMAL 0.1 mM reduced state 3 respiration (MOc3) in diabetic hearts compared to the Sham group (Sham 80.3 ± 14.4 vs. 50.1 ± 5.7 pmol O_2_/(s*mg)). RCR was reduced in the DiMAL 0.1 mM group in both non-diabetic (DiMAL 0.1 mM 1.52 ± 0.21 vs. Sham 2.15 ± 0.34 vs. IR 2.03 ± 0.23 vs. DiMAL 0.6 mM 2.23 ± 0.27, (p < 0.05)) and diabetic hearts (DiMAL 0.1 mM 1.52 ± 0.23 vs. Sham 2.03 ± 0.14 vs. IR 1.88 ± 0.23 vs. DiMAL 0.6 mM 1.95 ± 0.16, (p < 0.05)). Finally, diabetes was associated with an overall decrease in respiration as observed by state 3 respiration and RCR (ANOVA: p = 0.007 and 0.02).

### Mitochondrial enzyme activity

#### Isolated rat heart study

**Figure 8:**
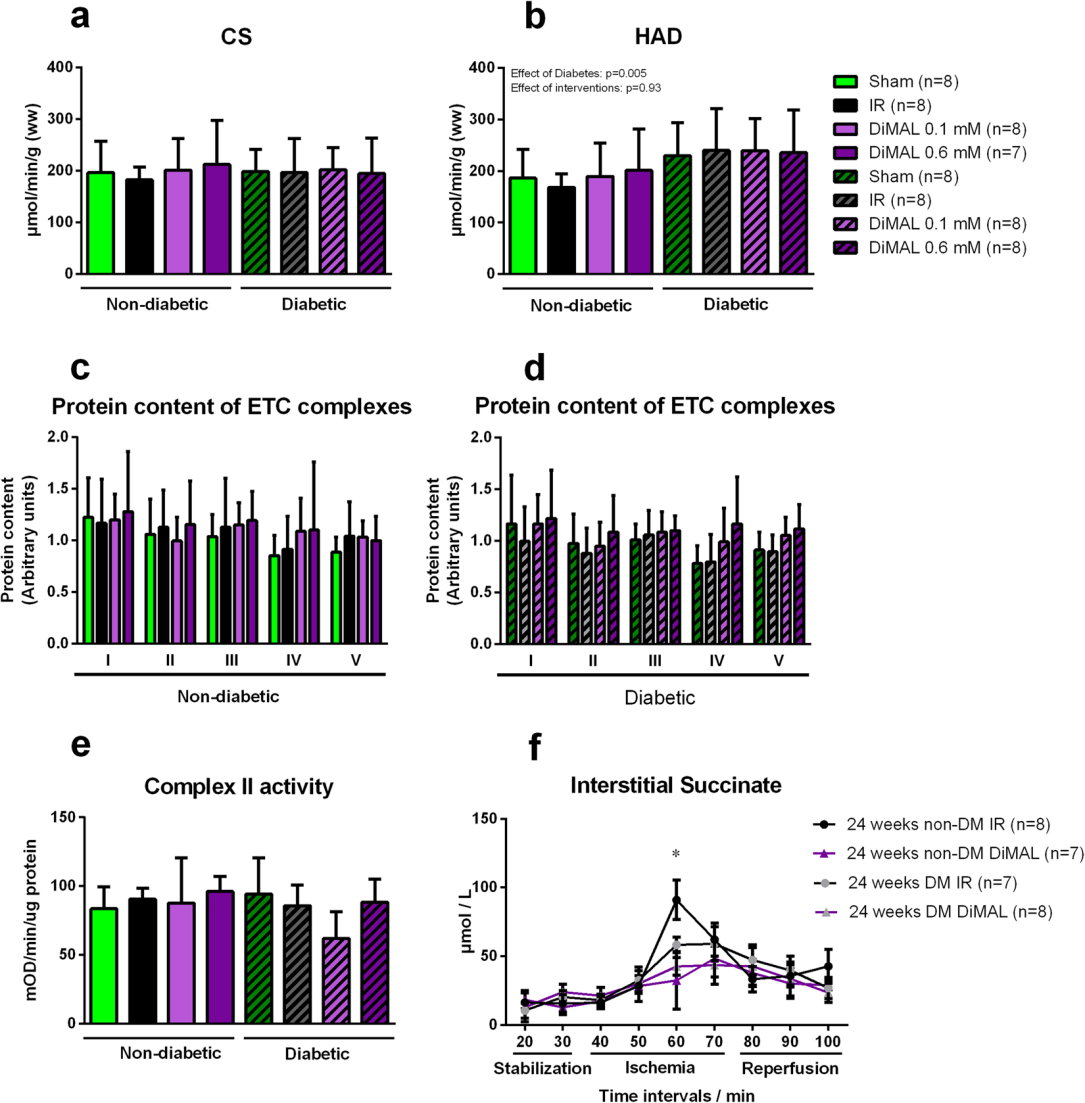
Isolated rat heart study - Mitochondrial enzyme activity. Enzyme activity of citrate synthase (CS) and β-hydroxyacyl-CoA dehydrogenase (HAD) (**a,b**). Below protein content of individual electron transport chain complex I-V in non-diabetic (**c**) and diabetic (**d**) heart tissue. At the bottom Complex II activity in non-diabetic and diabetic tissue (**e**) and interstitial succinate concentrations (**f**). IR: Ischemia reperfusion, DiMAL: Dimethyl malonate. Data are the mean ± SD. *P < 0.05 between indicated groups. P-values at the top indicate overall effect of diabetes and interventions by two-way ANOVA, respectively.

No overall difference in CS activity was seen between the diabetic and non-diabetic group. Citrate synthase (CS) activity did not differ between groups in the non-diabetic hearts (Sham 196 ± 61 and IR 182 ± 25 and DiMAL 0.1 mM 201 ± 61 and DiMAL 0.6 mM 212 ± 85 µmol/min/g (p = 0.82)) or the diabetic hearts (Sham 198 ± 44 and IR 196 ± 66 and DiMAL 0.1 mM 201 ± 44 and DiMAL 0.6 mM 194 ± 69 µmol/min/g (p = 0.99) (Fig. 8). β-Hydroxyacyl-CoA dehydrogenase (HAD) activity was not different between intervention groups in either the non-diabetic (Sham 187 ± 55 and IR 168 ± 26 and DiMAL 0.1 mM 190 ± 65 and DiMAL 0.6 mM 201 ± 80 µmol/min/g) or diabetic hearts (Sham 230 ± 64 and IR 240 ± 81 and DiMAL 0.1 mM 239 ± 63 and DiMAL 0.6 mM 236 ± 83 µmol/min/g). Diabetic hearts had a higher HAD activity than non-diabetic hearts (ANOVA p < 0.05).

### Mitochondrial protein content of ETC complexes and complex II activity

The protein content of the mitochondrial ETC complexes I-V in cardiac muscle did not differ between groups (Fig. 8) or between non-diabetic and diabetic cardiac tissue. Complex II activity in cardiac muscle was similar in all groups (Fig. 8).

### Interstitial succinate concentration

Diabetic IR hearts had statistically non-significantly lower succinate concentration than non-diabetic IR hearts (58 ± 14 vs. 90 ± 35 vs. µmol/L, p = 0.30) (Fig. 8). After 60 minutes of ischemia DiMAL reduced succinate concentrations in the non-diabetic group (90 ± 35 vs. 33 ± 42 µmol/L, p < 0.05). DiMAL also reduced interstitial succinate concentration in diabetic hearts but the difference was not statistically significant (43 ± 18 vs. 58 ± 14 µmol/L, p > 0.99).

## Discussion

The main finding of the present study is that inhibition of the SDH by administration of DiMAL protects the heart from IR injury in non-diabetic and diabetic animals. However, diabetes seems to reduce the sensitivity to DiMAL such that an increased dose is required to elicit a cardioprotective response. The DiMAL induced cardioprotection was associated with an improvement of post-ischemic mitochondrial function in non-diabetic animals but not in diabetic animals. Diabetes *per se* compromised mitochondrial respiration capacity, reflecting that mitochondrial dysfunction may explain reduced sensitivity to cardioprotective strategies in experimental models of diabetes. At a dose optimized through a dose-response experiment, a cardioprotective effect of DiMAL was present in human cardiac tissue from patients without diabetes, while cardiac tissue from patients with diabetes was not protected by the administered doses of DiMAL. In contrast to our experimental findings, mitochondrial function was not compromised in human cardiac tissue from diabetes patients and the absent effect of DiMAL was not associated with mitochondrial respiratory capacity.

Our results support previous findings of a cardioprotective effect by DiMAL^11,31,32^. SDH inhibition has become an attractive target in pharmacologic conditioning because it may interact with central mechanisms underlying IR injury^11^. Complex I is the major source of mitochondrial ROS production. During ischemia, the purine nucleotide cycle (PNC)^11^ and the MAS^27^ supply fumarate to Complex II, which acts in reverse to reduce fumarate to succinate. At reperfusion, oxygen supply is restored, and Complex II rapidly metabolizes the excess succinate in its forward direction to fumarate. However, a delay in the subsequent steps prevents further Complex III-V metabolism, when the mitochondrial membrane potential increases. As a consequence, electrons are forced in a reverse direction through Complex I generating large amounts of ROS. Excessive ROS production ultimately cause opening of the mitochondrial permeability pore complex and cell death^9^.

DiMAL is a competitive inhibitor of the SDH that binds to the succinate site of the enzyme^15^. DiMAL can be administrated prior to index ischemia to reduce succinate accumulation^11^ or during early reperfusion to inhibit succinate oxidation thereby limiting reverse electron transport and ROS production^32^. Our data demonstrate that pre-ischemic administration of DiMAL, mimicking preconditioning, is cardioprotective both in rats and in human cardiac tissue with an efficacy comparable to IPC. Remote IPC, postconditioning, hypothermia and cyclosporine A can induce the same level of cardioprotection as DiMAL^33,34^. The additive effect of hypothermia and DiMAL on infarct size was protective by distinct mechanisms. It is unknown if remote IPC, postconditioning or cyclosporine A modifies ROS production by attenuation of succinate production. Our data showed that the succinate/ROS pathway does not seem to be an inherent part of IPC, since IPC does not inhibit succinate accumulation during ischemia^16,27,35^ or succinate oxidation during early reperfusion^27,36^. These findings suggest that a combination of different therapeutic approaches could further protect against IR injury during acute myocardial infarction.

We found that an increased concentration of DiMAL was needed to reduce infarct size in diabetic compared to non-diabetic animals. This might be caused by changes in the amount or activity of SDH, since DiMAL is a competitive inhibitor of SDH. We did not observe any change in protein content of any of the complexes of the ETC or differences in complex II activity between groups. The finding indicates that the need for higher concentration of DiMAL may be caused by factors other than protein content and complex II activity, e.g. metabolic rate control or substrate selection beyond the mechanisms traditionally claimed to explain the glucose-fatty-acid cycle^37^. Using dosages optimized to non-diabetic trabeculae, we found no protection by DiMAL in patients with diabetes. The dosage used in human tissue was chosen from the dose-response relationship in non-diabetic tissue and not customized to the diabetic phenotype. The optimized dosage for tissue from patients without diabetes was 10 fold higher than in the experimental setting. This is due to difference in drug delivery. In the isolated hearts DiMAL was delivered directly though the coronary vessels, whereas the atrial trabeculae were superperfused, demanding a higher concentration to achieve effect. Taking the experimental data into consideration, it seems plausible that the dosage was insufficient because diabetic animals needed a higher dose than non-diabetic animals to achieve protection. Due to low inclusion rates of patients with diabetes, it was not possible to evaluate the effect of a higher dosage in the current study, so we cannot exclude that a higher threshold might be present in humans as previously demonstrated in the diabetic rats^38^. Our data suggest that the therapeutic window for SDH inhibition is narrow and this may be a translational challenge. We found that even small changes in concentrations of DiMAL could abrogate the protection, supporting previous observations in mice that revealed a toxic effect of DiMAL on hemodynamic performance under normoxic conditions^31^. The respiratory capacity of complex I was severely compromised by the high DiMAL dosage in non-diabetic rats (DiMAL 0.6 mM) reflecting the narrow therapeutic window of DiMAL. The deleterious effect may be explained by an irreversible, detrimental effect of combining excessive SDH blockade with IR injury, which may inhibit restoration of normal respiration during reperfusion. No improvement in mitochondrial respiration was observed in human trabeculae treated with DiMAL or IPC indicating that cardioprotection may be achievable without measurable modulation of post-ischemic mitochondrial function.

Preserved post-ischemic mitochondrial function is important for cellular function and survival^9,39^. Our data confirmed that diabetes has an inherent deleterious effect on overall mitochondrial respiration in our experimental setting, which adds to the impaired respiratory capacity following ischemia. We found that administration of 0.1 mM DiMAL to non-diabetic hearts tended to improve complex I-linked respiration reflecting reduced injury to the complex. This improvement in complex I-linked respiration correlates with the current hypothesis that DiMAL limits succinate accumulation, reverses electron transport to complex I and ROS-mediated injuries^11^.

DiMAL reduced accumulation of interstitial succinate during ischemia in non-diabetic and diabetic rat hearts. Diabetic hearts seemed to have an inherent ability to reduce succinate accumulation compared to non-diabetic hearts. Reduced succinate accumulation during an ischemic event has previously been reported to provide cardioprotection^16^. We found no correlation between reduced succinate accumulation and cardioprotection since IR exposed diabetic hearts and DiMAL treated non-diabetic hearts did not have lower infarct sizes compared to non-diabetic IR hearts. The missing correlation between succinate buildup during ischemia and cardioprotection has previously been reported in the setting of ischemic preconditioning^40,41^, so the role of succinate in IR injury remains unresolved.

Diabetic tissue from rat hearts showed elevated levels of HAD activity corresponding to an elevated fatty acid metabolism possibly caused by a reduced ability to metabolize glucose. However, *ex vivo* rat heart fatty acid oxidation was reduced indicating that other steps in the fatty acid metabolism may be impaired in the diabetic hearts. IR reduced fatty acid oxidation and treatment with DiMAL did not improve the respiratory capacity in spite of its cardioprotective effect. This finding may simply reflect that the isolated rat hearts only received glucose during the perfusion protocol or that fatty acid metabolism may be less important compared to glucose metabolism in the protection against IR injury.

CS activity did not differ between non-diabetic and diabetic hearts indicating a similar level of mitochondrial content. Diabetes can both up- and down-regulate mitochondrial content over the course of disease progression^42^. Our findings may suggest that the diabetic disease model had not yet developed major changes in mitochondrial content and support that the respiratory findings are mainly driven by changed respiratory capacity rather than the number of mitochondria.

The fact that co-morbidities such as diabetes and co-medication including glucose lowering therapy modify the sensitivity to cardioprotection and consequently changes the concentration required to elicit cardioprotection increases the complexity of applying the concept into clinical use. This challenge is reflected by the discrepant findings between our experimental and human studies. This is probably not a specific characteristic for DiMAL but for all modulators of the mitochondrial function. Future studies should focus on safe delivery of ETC modifiers to the target area in the optimal concentration with absent systemic effect. A recent study addressed this problem and demonstrated the potential of intracoronary administration of DiMAL in pigs^32^. While intracoronary administration increases the therapeutic value of the drug, it will most likely be a delicate balance to achieve optimal effect without introducing side effects.

An alternative and safer way to modulate mitochondria may be utilization of a physiological inhibitor rather than a synthetic inhibitor, which cannot be metabolized in the cell. Oxaloacetate and malate are tricarboxylic acid cycle intermediates with strong inhibitory effect on SDH^15^, similar to that of DiMAL. It may be possible to achieve a protective effect by increasing the levels of e.g. oxaloacetate. Protection by oxaloacetate has been tested in rats in a focal brain ischemic model with evidence suggesting a neuroprotective effect^43^. The concept is further supported by the possibility to induce cardioprotection through MAS inhibition by aminooxyacetate (AOA) during late ischemia^16^. AOA inhibits the aspartate amino transferase, which uses oxaloacetate as a substrate and it is possible that the protection works though upregulation of oxaloacetate^16^. Finally, short cycles of IPC have been shown to increase malate, the precursor to oxaloacetate, indicating a possible involvement of these tricarboxylic acid cycle intermediates in the protection of IPC^16^.

The current study has limitations. First, we used a ZDF rat model to examine the impact of mature diabetes on myocardial IR injury. The ZDF rat is mutated in the leptin receptor resulting in a diabetic phenotype similar to the human. Because the leptin receptor is defect, the animals have high circulating levels of leptin, which may activate protective pathways^44^. However, the ZDF model is considered a valid model for type 2 diabetes because of the similarities with the human phenotype. Second, the baseline characteristics of the animals were consistent with a diabetic phenotype similar to humans with elevated fasting blood glucose, insulin, cholesterol, triglycerides and FFA. However, compared to previous studies, the animals in the diabetic groups had lower levels of circulating glucose concentrations^22^. Only animals randomized to the DiMAL 0.1 mM groups had blood glucose levels similar to previous observations^22^. However, all animals in the diabetic groups had elevated blood glucose and insulin levels and a phenotype consistent with a metabolic syndrome. Third, we used an isolated rat heart model to evaluate the cardioprotective effects. Isolation of the heart excludes the influence of systemic and humoral factors during myocardial IR. Our circulation buffer did not contain insulin or FFA and does not resemble normal physiology. Fourth, we used permeabilized myocardial fibers for evaluation of mitochondrial respiration. This is a validated method to investigate the mitochondria *in situ*, and seems to be more physiologically correct than isolated mitochondria^45^. Even so, it does not reflect physiological conditions, which may limit the translation into the clinical setting. Fifth, tissue from the right atrial trabeculae may not reflect the function, physiology and metabolism of the left ventricle. Nevertheless, similarities between atrial and ventricular tissue have been demonstrated^46,47^ and the right atrial appendage remains the most ethically applicable method to examine human myocardium. Tissue was transported from the operating theater to our laboratory in an oxygenated buffer with a transport time below 5 minutes. It cannot be excluded that partial hypoxia might affect the tissue prior to suspension in the atrial strip model. Finally, we acknowledge the lack of insight into ROS production and injury, which limits the mechanistic understanding and interpretation of our data.

In summary, pre-ischemic inhibition of the SDH by DiMAL protects the non-diabetic and diabetic rat heart from IR injury. The protection can be translated into human cardiac tissue from patients without diabetes and is as effective as IPC. In our animal model, mature diabetes alters the metabolic phenotype that resulted in higher resistance to SDH inhibition and requirement for a higher dosage to induce cardioprotection. Accordingly, DiMAL treatment with an optimized dosage through dose-response correlation did not protect the diabetic human myocardium. The protection is associated with improved post-ischemic mitochondrial function in our isolated heart model, but not in the human atrial trabeculae. Conceptually, SDH inhibition may provide a pharmacological target to counteract IR injury. However, synthetic agents have narrow therapeutic range and efficacy may be influenced by diabetes.
